# Supplementary material for: An evolution-based high-fidelity method of epistasis measurement: Theory and application to influenza
Source: PLoS Pathog. 2021 Jun 21;17(6):e1009669. doi: 10.1371/journal.ppat.1009669 (PMC8248644; doi:10.1371/journal.ppat.1009669)
Supplement: S1 Text — (PDF) [file ppat.1009669.s001.pdf]

# Supporting Information

"An evolution-based high-fidelity method of epistasis measurement: theory and application to influenza" by Gabriele Pedruzzi and Igor M. Rouzine

*Sorbonne Université, Institute de Biologie Paris-Seine, Laboratoire de Biologie Computationelle et Quantitative LCQB, F-75004, Paris, France*

### S1 Text. Derivation of UFE for the closed square topology

The topology of the network consists from $L$ isolated closed squares (S1A Fig). In this case, Eq. 5 takes the form

$$-W/s_0 = k_1 + 2k_2' + 2k_2(1-E) + k_3(3-4E) + k_4 4(1-2E) \qquad (1)$$

where $k_i$ are the numbers of five types of configurations: single, opposing double, triplet, full square, respectively (S1A Fig). Each configuration $i$ has $n_i = 1, 2,$ or $4$ symmetric transformations. Eq. 6 for entropy takes the form

$$S = \log\left[C_L^{k_4} C_L^{k_3} 4^{k_3} C_L^{k_2} 4^{k_2} C_L^{k_1} 4^{k_1} C_L^{k_2'} 2^{k_2'}\right] \qquad (2)$$

We introduce the probability of configuration $i$

$$f_i = \frac{k_i}{L}$$

Putting derivative of $S$ in $k_i$ in Eq. 2 to zero, we get

$$0 = df_1 \log\frac{4}{f_1} + df_2' \log\frac{2}{f_2'} + df_2 \log\frac{4}{f_2} + df_3 \log\frac{4}{f_3} + df_4 \log\frac{1}{f_4} \qquad (3)$$

This maximum is conditioned by the fact that fitness defined by Eq. 1 is fixed, hence

$$-df_1 = 2\, df_2' + 2(1-E)df_2 + (3-4E)df_3 + 4(1-2E)df_4 \qquad (4)$$

Here all $df_i$ in the right-hand side of Eq. 4 are independent differentials. Substituting Eq. 4 into 3 and demanding that all coefficients at these independent differentials are zero, we can express all $f_i$ in terms of $f_1$ as

$$\frac{f_2'}{2} = \left(\frac{f_1}{4}\right)^2, \quad \frac{f_2}{4} = \left(\frac{f_1}{4}\right)^{2(1-E)}, \quad \frac{f_3}{4} = \left(\frac{f_1}{4}\right)^{3-4E}, \quad \frac{f_4}{4} = \left(\frac{f_1}{4}\right)^{4(1-2E)} \qquad (5)$$

Without the loss of generality, we can consider an interval $0 < E < 1/2$, because below 0, as we shall see, indirect interactions do not emerge. Above $E = 1/2$, all

these values diverge due to over-compensation, i.e., mutations accumulate without limit. We also assume that the system is not too far from the best-fit sequence, $f_1 \ll 1$. Under these assumptions, the following inequalities apply

$$f_2' \ll f_2, \quad f_1 \gg f_2 \gg f_3 \tag{6}$$

Based on Eqs. 5 and $f_1 \ll 1$, the probability of a 4-site cluster, $f_4$, is ordered with respect to the other probabilities depending on subdivision of this interval of $E$

$$\begin{array}{cc} E < \frac{1}{4} & f_4 \ll f_3 \\ \frac{1}{4} < E < \frac{1}{3} & f_3 \ll f_4 \ll f_2 \\ \frac{1}{3} < E < \frac{3}{8} & f_2 \ll f_4 \ll f_1 \\ E > \frac{3}{8} & f_4 \gg f_1 \end{array} \tag{7}$$

We now calculate the frequencies of haplotypes for 2 sites locating on the opposite corners of a square, which corresponds to indirect interaction (S1B Fig). By adding all possible configurations that can produce 11 at these two sites, we obtain

$$f_{11}^{ind} = \frac{f_2'}{2} + \frac{f_3}{2} + f_4 \tag{8}$$

$$f_{10}^{ind} = \frac{f_1}{2} + \frac{f_2}{2} + \frac{f_3}{4} \tag{9}$$

Now we will calculate three-way haplotype by putting fixed 0 at a third site (S1C Fig). Now, a part of configurations are eliminated from Eqs. 8 and 9 and we get smaller values

$$f_{110}^{ind} = \frac{f_2'}{2} + \frac{f_3}{4} \tag{10}$$

$$f_{100}^{ind} = \frac{f_1}{2} + \frac{f_2}{4} \tag{11}$$

We also can calculate a 4-way haplotype for the indirect interaction when two 0s are added (S1D Fig)

$$f_{1100}^{ind} = \frac{f_2'}{2}, \qquad f_{1000}^{ind} = \frac{f_1}{4} \tag{16}$$

Now we repeat the same procedure for two sites located on one side of a square, which corresponds to direct interaction (S1E Fig and S1F Fig)

$$f_{11}^{dir} = \frac{f_2}{4} + \frac{f_3}{2} + f_4 \tag{12}$$

$$f_{10}^{dir} = \frac{f_1}{4} + \frac{f_2'}{2} + \frac{f_2}{4} + \frac{f_3}{4} \tag{13}$$

$$f_{110}^{dir} = \frac{f_2}{4} + \frac{f_3}{4} \tag{14}$$

$$f_{100}^{dir} = \frac{f_1}{4} + \frac{f_2}{4} \tag{15}$$

Using our assumption $f_1 \ll 1$ again, we have $f_{00} \approx 1$, and the definition of UFE, Eq. 1 in the main text, takes a simplified form

$$UFE_{ij} = 1 - \frac{\log(f_{11})}{\log(f_{10}^2)} \tag{16}$$

where we used $f_{10} = f_{01}$ due to the symmetry of the topology. Using strong inequalities in Eqs. 6 and 7 and substituting Eqs. 10 to 16 into Eq. 17, we obtain the values of all types of UFE in different intervals of $E$ shown in S1 Table. The dependence of each kind of correlation measure UFE on epistatic strength $E$ is plotted in Fig. S2.

We can draw several conclusions, as follows:

i) Indirect interaction is absent at small $E$, $E < 1/4$. By induction, it is also absent at negative $E$, where large clusters creating indirect interaction are very rare. Direct $UFE$ equals $E$, as if the pair is isolated epistatically [46].

ii) At large $E > 1/3$, direct and indirect pairwise correlation have exactly the same magnitude, and direct UFE exceeds the value of $E$. The intuitive reason for these results is that direct and indirect UFEs are both determined mostly by 4-allele clusters, which are numerically dominant over the smaller clusters (Eq. 7).

iii) At large $1/2 > E > 1/3$, the addition of 0 at a third site makes direct and indirect correlation distinct from each other (and smaller).

iv) In this case, the addition of another 0 at the remaining site kills indirect correlation.

Therefore, the 3-way correlation method can, in principle be used to tease the direct and indirect interactions apart. However, it remains of the same order of magnitude as direct interaction, especially if $E$ is close to full compensation point ($E = 1/2$), and because the magnitude varies broadly between pairs in real biological systems, this difference may be not enough for reliable detection. Hence, the best way is to add another 0 and measure 4-way haplotypes. This trick eliminates indirect interactions completely, $UFE_{ind}^{00} \equiv 0$, in the entire interval of $E$.

Intuitively, we interrupt both “detours” along interacting pairs connecting the two loci of interest.

In the case of the most general topology with many loops, this simple example leads to the generalization that the number of the additional zeros required to kill an indirect interaction is equal to the number of directions in which a detour can occur from a site of the pair. For example, a site of a suspected pair has six epistatic partners, and two of them start a detour to the other site of the pair. In this case, we would need to add two 0s. Hence, one needs iteratively to add extra 0s and see if anything has changed. We did not have to use this procedure for virus protein data in Fig. 2, because the detected network is almost a tree already after the three-way test.
