# Supplementary figures and images for: An evolution-based high-fidelity method of epistasis measurement: Theory and application to influenza
Source: PLoS Pathog. 2021 Jun 21;17(6):e1009669. doi: 10.1371/journal.ppat.1009669 (PMC8248644; doi:10.1371/journal.ppat.1009669)

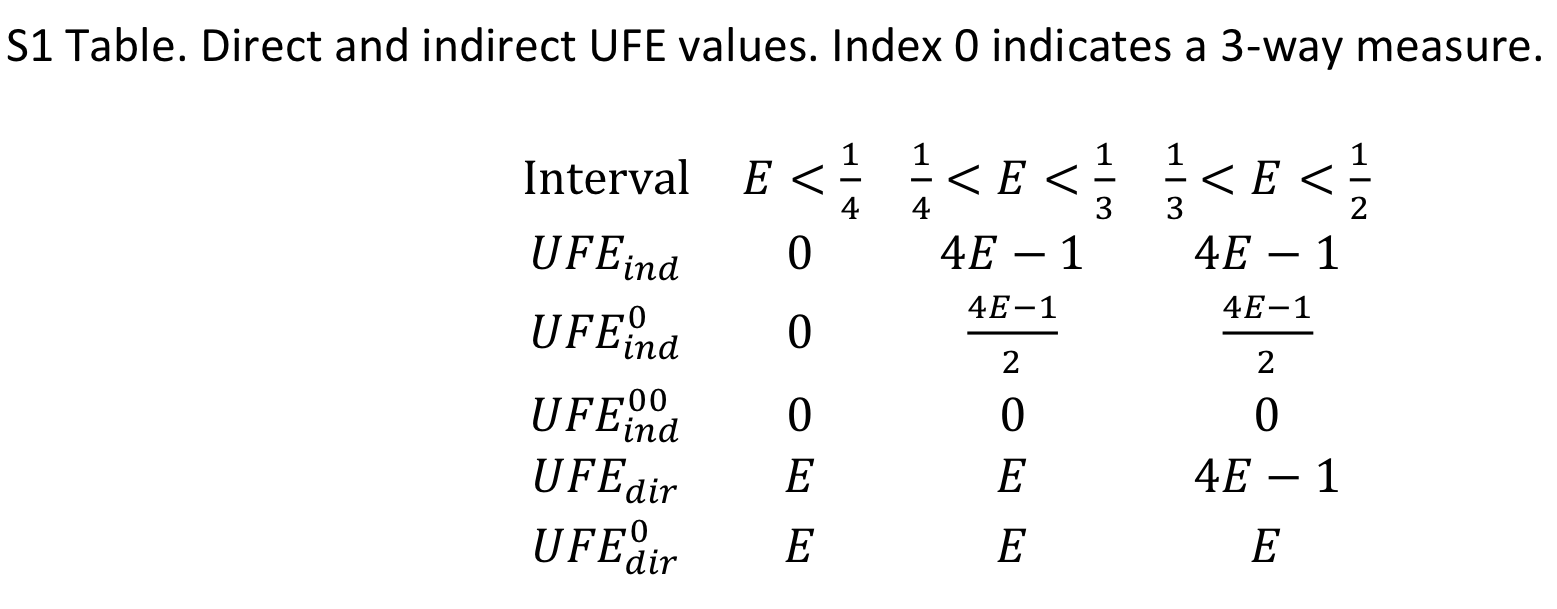

Supplement: S1 Table — Index 0 indicates a 3-way measure. (TIFF) [file ppat.1009669.s002.tiff]

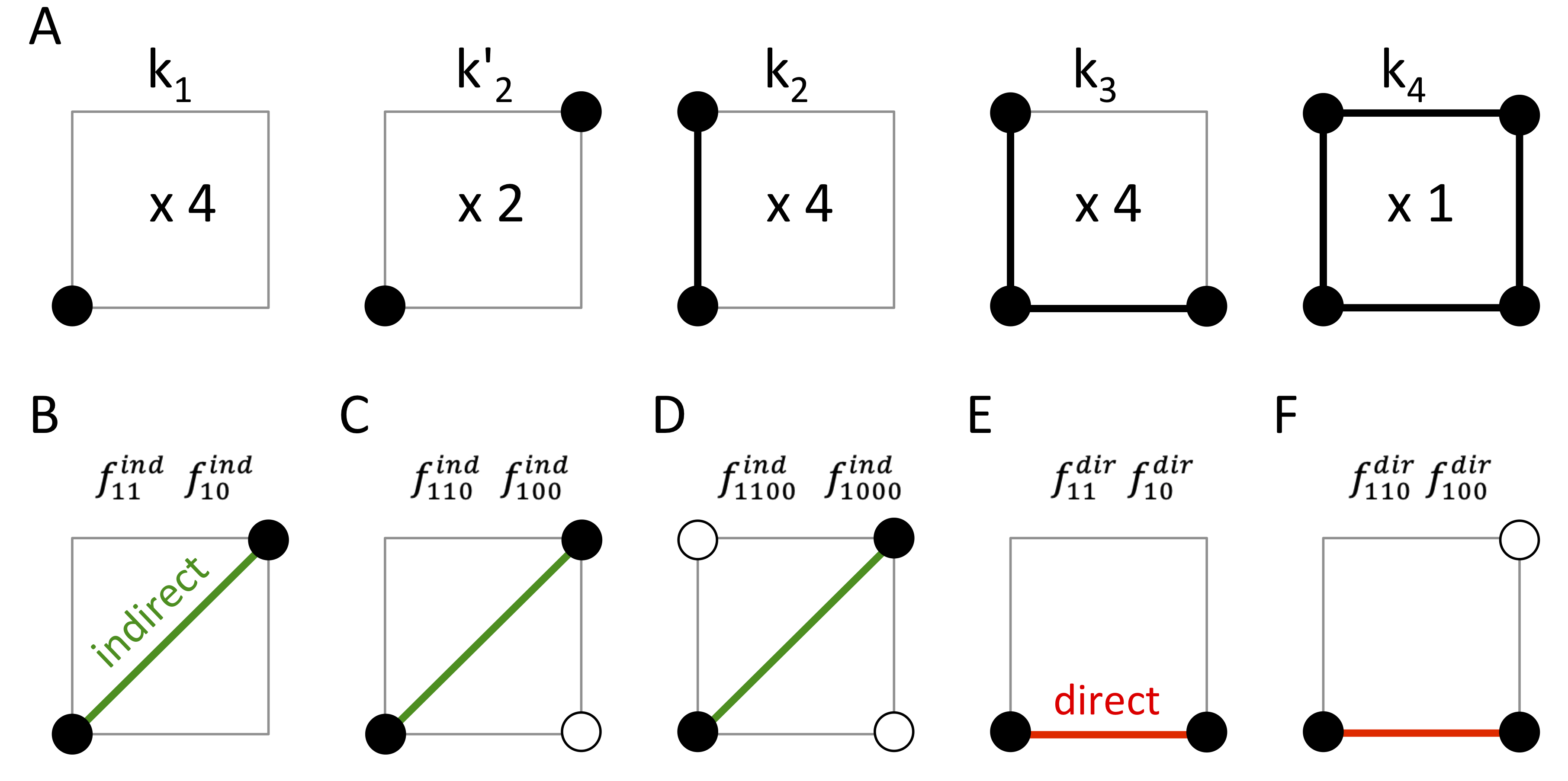

Supplement: S1 Fig — A) Possible configurations and their symmetry. B) Indirect interaction pairwise. C) Indirect interaction three-way, with a fixed zero at a node. d) Indirect interaction with two fixed zeros. E) Direct interaction pairwise. F) Direct interaction three-way. (TIFF) [file ppat.1009669.s003.tiff]

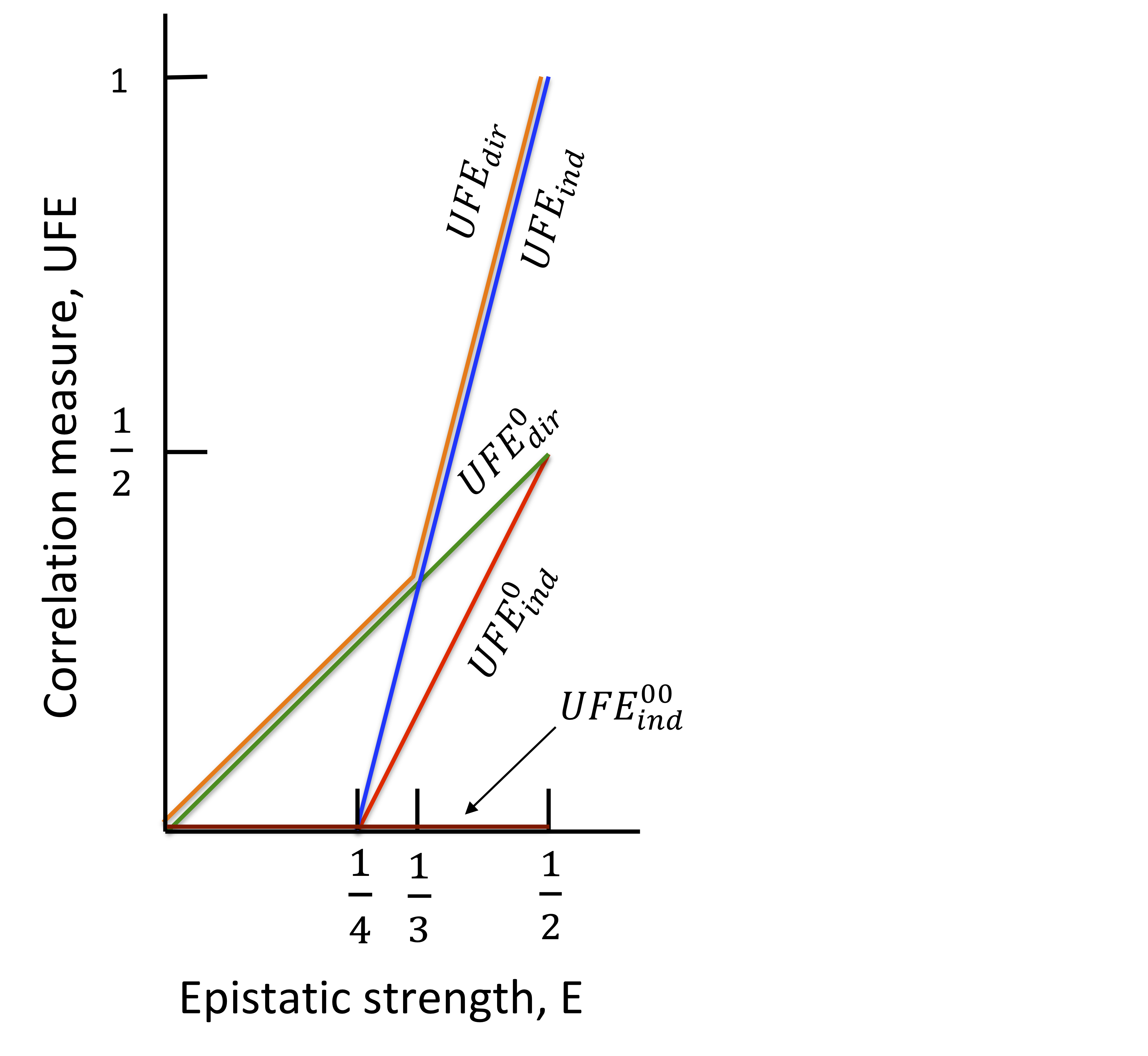

Supplement: S2 Fig — (TIFF) [file ppat.1009669.s004.tiff]
