# Supplementary material for: An evolution-based high-fidelity method of epistasis measurement: Theory and application to influenza
Source: PLoS Pathog. 2021 Jun 21;17(6):e1009669. doi: 10.1371/journal.ppat.1009669 (PMC8248644; doi:10.1371/journal.ppat.1009669)
Supplement: Attachment — Submitted filename: Rebuttal.pdf [file ppat.1009669.s006.pdf]

Reviewer #1:

This study is valuable in its approach of using population genetics theory to devise a method of detecting epistasis among sequences sites. The approach is rigorous and well defended.

I thank the Reviewer for the positive opinion. Line numbering below is not sequential between pages and is given for the marked copy with traced changes.

p. 11 - The explanation for using the triple-site haplotype method should be made clearer at this point, even though it is explained in the Methods section.

I added the explanation in two places:

Lines 264-267:
The basic idea behind this method is that demanding a majority allele at a neighbor site of a measured pair of sites, cuts the indirect detours around the pair that create false-positive interaction. In other terms, the additional condition splits the genome into independent blocks.

Line 562-569:
The idea of the procedure is based on the fact that false-positive links are created by "detours" around pairs of sites, such as chains of indirect epistatic interactions or sited linked due to the common phylogenetic origin (red and blue curves in Fig. 1B). To break up the association created by detours, we demand that a neighbor site of the site pair of interest is 0 (better-fit, wild-type) and recalculate the correlation. If that neighbor site happens to be at the most important detour, this condition will break up or, at least, decrease the indirect correlation. Direct interactions are affected by such an additional condition to a smaller extent (if at all).

p. 12 - Although it seems a reasonable assumption, there should be some justification for assuming an epistatic network for NA.

We do not assume. We infer it. We detect true interactions and measure their magnitude. I made it clear now:

Lines 276-349:
Using 8000 influenza sequences obtained from various geographic locations, both before and after the pandemic of 2009, we infer the epistatic network in a surface protein, neurominidase

Lines 351:
Our aim is to identify the primary and secondary mutations that allowed the new strain to outcompete the old strain.

p. 15 - Surely there is some empirical evidence for this. Is there any empirical evidence that the 15-20 compensatory mutations are actually compensatory?

I am not aware of any such evidence, even though we looked for it in the literature. Thus, our work makes a testable prediction. In the revised version, I state:

Line 981-987:

We did not find any empiric evidence in the literature for or against the inferred network of compensatory mutations. Hence, we have a testable prediction for a future experimental test. Primary site 248 can be predicted by simple alignment and is well studied experimentally. Its compensatory sites, however, are impossible to detect without our method, due to strong linkage noise. It would be very useful to compare these predictions with the results of deep mutational scanning [52, 53], which we hope will be available in the future.

Also, the identification of the primary site could presumably be made by simply comparing aligned sequences from before and after the pandemic. It would be useful to comment on this.

That is correct, and I made a note in Line 983:

Primary site 248 can be predicted by simple alignment and is well studied experimentally.

More generally, the method has been tested using compensatory epistasis, but is there any evidence it works with other forms of epistasis?

Yes. I added an explanation in Lines 675-679:

The formalism applies at negative values of E as well, but we focus on positive E, which case is termed "diminishing returns epistasis". The reason for this choice is strong effects of epistasis and strong indirect interactions in this region. The analytic derivation in below applies at any $E < 1$ and below the full compensation point, which covers all basic types of epistasis.

This is an editorial comment. There are minor errors of sentence structure or typographical errors throughout the manuscript. These seem typical of writers that are not native English speakers and could be easily corrected by a native speaker or by the journal.

The manuscript has been copyedited in depth.

Reviewer #2:

The authors propose a version of the linkage-disequilibrium test for detecting pairwise epistasis in sequence data. A major obstacle in detecting epistatic pairs is that many other pairs are also at linkage disequilibrium, due to hitchhiking or due to indirect interactions. The authors propose a procedure for reducing this noise which relies on estimating conditional LD, conditioned on a potential confounding site being at its wildtype state. They apply this approach to identifying epistatic networks in influenza H1N1 neuraminidase data and find that a previously known site 248 near the active pocket is involved in many interactions.

The authors ask an important question. Their core idea makes sense and is promising.

I thank the Reviewer for appreciating the challenge and the promise. Line numbering below is not sequential between pages and is given for the marked copy with traced changes.

However, I see two major issues with this manuscript, which I will discuss in detail below. The first problem is that the authors have not demonstrated how well their method works. In what regimes is it expected to work? When does to fail? The second problem is the organization of the manuscript and a lack of clarity in many places, which make reading it difficult and at times frustrating, even for a specialist.

I addressed these issues below.

1. INSUFFICIENT EVIDENCE. The idea to detect epistasis using linkage disequilibrium is not new. It is clear why it works in sexual populations or in a system at mutation-selection balance. Here, the authors argue that essentially the same idea can also work in rapidly adapting asexual populations, such as the influenza virus. But why and when it works is unclear. Figure 2 convinces me that it works sometimes.

The main evidence is in the general analytic derivation in *Methods*, which is the central finding of the paper. I have added the missing explanation and the intuitive interpretation in three places, as follows:

Lines 263-271

The new technique is based on the use of three-way haplotypes. The basic idea behind this method is that demanding a majority allele at a neighbor site of a measured pair of sites, cuts the indirect detours around the pair that create false-positive interaction. In other terms, the additional condition splits the genome into independent blocks.

The high fidelity of this detection technique is demonstrated below by using two parallel methods: analytic derivation for a simple network topology and Monte-Carlo simulation. The analytic derivation makes no assumptions about the range of E or sign or epistasis. The simulation example considers compensatory epistasis, $0 < E < 1$, but the technique applies at any value of E, including negative epistasis.

Lines 568-575:

The idea of the procedure is based on the fact that false-positive links are created by "detours" around pairs of sites, such as chains of indirect epistatic interactions or sited linked due to the common phylogenetic origin (red and blue curves in Fig. 1B). To break up the association created by detours, we demand that a neighbor site of the site pair of interest is 0 (better-fit, wild-type) and recalculate the correlation. If that neighbor site happens to be at the most important detour, this condition will break up or, at least, decrease the indirect correlation. Direct interactions are affected by such an additional condition to a smaller extent (if at all).

Line 666-668:

Analytic results. In Methods, we show analytically that $min(UFE_{ij0}) \ll min(UFE_{ij})$ for indirect interactions and that this condition can be used to eliminate indirect interactions analytically, in the general form.

But I think for this paper to have impact, the authors need to provide at least some guidance as to when the method works and when it fails. For this method to work, the epistatically

interacting sites must be at minimum simultaneously polymorphic. The triple-site method requires three sites to be polymorphic at the same time. The genetic diversity of a population depends on the population genetic parameters, as the authors are well aware. For example, if the population evolves in the successional mutation regime, the UFE statistic cannot be evaluated and this method will not work. But it is not clear to me whether simply being in the concurrent mutation regime is sufficient. The authors heavily rely on their earlier paper (Pedruzzi et al, PLoS Comp Biol 2018), but I could not find a clear delineation of different parameter regimes.

I have added the necessary conditions:

in *Results,* Lines 668-673

In the analytic derivation, we assume that the system is under directed selection and in the multiple-mutation regime (the traveling wave regime), which takes place if $\log(NU) \gg \log(s/U)$ [12]. In this regime, selection sweeps at many sites overlap in time and interfere with each other [7]. In this case, a quasi-equilibrium condition is met (*Methods*). We also assume that the population is far from mutation-selection-drift steady state, so that deleterious mutation events are negligible.

and Methods, Lines 1169-1217.

Main approximations. We assume that the system is under directed selection and in the multiple-mutation regime (traveling wave regime), which takes place if $\log(NU) \gg \log(s/U)$ [12]. In this case, we have interfering selection sweeps occurring at many sites at once. We are far from mutation-selection-drift equilibrium, so that reverse (deleterious) mutations are negligible. In a broad parameter range, an adapting population can be represented by a slowly-moving, narrow peak in fitness coordinate [7, 9, 12-14]. Evolution is slow, because the limiting factor is the addition of a rare beneficial mutation established within a highly-fit genetic background [7, 12]. Because the fitness distribution moves slowly, the entropy (the log number of possible sequences given fitness) of the mutation distribution over genomes has enough time to reach its current maximum, restricted by the current average fitness of the population. This situation is called "quasi-equilibrium". At each moment, each fitness class has enough time to reach the most probable, most chaotic state given its fitness. Previously, we verified the validity of quasi-equilibrium in a broad range of parameters and initial conditions after time ~ 1/<*s*> [44, 58].

Ideally, I would like to see a phase diagram in the U, N and s space for a some simple epistatic model like the "double arches" showing where the UFE statistic between a specific pair of sites correlates well with the true value of E, where it does not correlate and where it cannot be evaluated.

Ideally, I agree. The problem is, we cannot calculate the full phase diagram yet. It is very hard. We have only the conditions above.

I stated it as a future goal in *Discussion*, Lines 1060-1064:

Our aim here was to propose the first method that can, in principle, isolate linkage from epistasis. Our analytic derivation, under the stated conditions, demonstrates that the method works in a broad range. The future task will be to find the full region of the applicability of our method in terms of N, s, U, and the number of sites L. We hope to address the upper limit on L in the future work.

I also added a qualifier "proof-of-principle" to the Abstract.

The second issue that I see is that the ability to detect epistasis using this method may depend on the structure of the epistatic network. Figure 2 shows that it works for the simplest possible structure, the "double arches". We do not have a great picture of how epistatic networks look like within actual proteins. But I think evaluating the performance of this method for some more complex networks would be necessary.

A very good idea. I have created a new Supplemental Information with an analytic derivation and two figures for a more complex topology. In the new Results, I discuss it in lines 680-706.

The third issue that I am concerned about is how the amount of data and the sampling protocol might affect method's performance. Most sites that carry beneficial mutations, e.g., in influenza, are polymorphic only transiently. This presumably means that the proposed test has to be applied to sequences sampled within a certain time window. So, how should one choose this window? And how sensitive is the method to this choice?

We explained now, Lines 765:

We considered only the sites that were strongly polymorphic (>5%) sometimes in the window of 5 years, during which time they contribute to the calculated correlation measures.

I am guessing me that the down-weighing that the authors did in their analysis of the influenza data set address this issue. But that was not clearly explained and seems ad hoc. In any case, this seems like an important issue and should be addressed directly in the paper.

....As mentioned above, the down sampling procedure is not part of their method, at least as described, and so its purpose has to be clearly explained.

We explained it in the revised text better (Lines 770-782):

In order to compensate for unequal sampling from the pre-pandemic and pandemic strain, the more abundant sequences with mutation frequency per genome less than a preset value, $f < d_v$, were randomly sampled and down-weighted by a coefficient, D_w , ranging from 5% to 50%. This procedure was done to balance the number of sequences sampled between the two strains. To obtain the average pairwise haplotype frequencies, f_{ij} we repeated the resampling multiple times.

Next, we followed the procedure described above (Fig. 1C-F) to infer the intra-protein network of interactions (Fig. 2). The dependence of results on (d_v, D_w), which infers between 15 and 22 compensatory sites, originates from the unequal presentation of local subpopulations in the database. We observe that slightly different weighting gives similar results (Fig. 2C to 2E) within a plateau region in Fig. 2F. Only if one strain is strongly overrepresented, the network disappears (Figs. 2A and 2B). Below, we choose the network variant shown in Fig 2D as the "golden middle" of the set.

Also, how much data is actually needed in practice to reliably estimate the conditional UFE?

Explained in Lines 720-724:

The amount of data must be sufficiently high to ensure that each three-site haplotype entering UFE be represented by a large number of sequences, with their inverse square root being the relative error of the haplotype frequency. In our case, this condition was fulfilled by setting cutoff at allelic frequency $f > 5\%$. We downloaded 8440 sequences of NA from a public database (https://www.fludb.org).

2. CLARITY. There are several major clarity issues. Minor issues are listed below. I think the authors made a poor choice in how to introduce their approach. First, the second half of the intro where the authors essentially summarize part of their previous paper (Pedruzzi et al, 2018) is not understandable on its own without actually reading the supplement of that paper. Second, this theory is only relevant in so far as it provides sort of an explanation why the LD-based method might work in adapting asexual populations. But it is actually unnecessary for understanding the basic idea behind the UFE statistic, which is just a way to detect LD. Third, this explanation is not even satisfactory because it remains unclear when the assumptions made in their model actually hold (see above). So, the reader becomes very confused and skeptical before getting to the Results

I agree. I have removed the re-derivation of our previous results from the Introduction.

Fourth, the UFE statistics are not introduced in the Results section. I think they should be introduced, since it is the central contribution of this methodological paper.

Done, see Eqs. 1 and 2.

I found the analytical results for the double-arches model quite helpful. I think the authors should consider discussing these results in the main text.

Added in Lines 666-705:

Analytic results. In *Methods,* we show analytically that $min(UFE_{ij0}) \ll min(UFE_{ij})$ for indirect interactions and that this condition can be used to eliminate indirect interactions analytically, in the general form. In the analytic derivation, we assume that the system is under directed selection and in the multiple-mutation regime (the traveling wave regime), which takes place if $\log(NU) \gg \log(s/U)$ [12]. In this regime, selection sweeps at many sites overlap in time and interfere with each other [7]. We also assume that the population is far from mutation-selection-drift equilibrium, so that deleterious mutation events are negligible.

We also repeated this derivation for a more complex topology of closed squares (*Supplement*, Fig. S1A). Here indirect interaction occurs between the opposite corners of the square, and direct interaction between the sites of one side. This topology is more complex, because it has a loop, and there are two paths connecting the opposite corners. The results show that at $E > 1/3$, the magnitude of direct and indirect correlations is the same. The 3-way correlation method decreases indirect correlation to a larger degree, which can be used to tell the direct and indirect correlations apart (Fig. S1B, Table S1). Thus, the 3-way method is robust with respect to a topology. The difference from the double-arch case is that indirect correlation does not disappear completely and remains of the same order of magnitude as the direct interaction. This is especially true if E is close to the full compensation point (in this case, $E = 1/2$). Because, in real biological systems, the value of E varies broadly across pairs, such a difference may be not enough for the reliable detection of the true links demonstrated above for a loop-less topology. The natural way to address this issue is to add another 0 and measure a 4-way correlation to interrupt both equal detours connecting the two points. This trick, indeed, kills indirect interactions completely in the entire interval of E, as given by $UFE_{ind}^{00} \equiv 0$ (Fig. S2, Table S1).

For the general topology with many loops, the number of the additional zeros required to kill an indirect interaction completely is equal to the number of directions in which a detour can occur (the connectivity parameter). For example, if a chosen site of interest has six neighbor sites with strong correlation (direct or indirect, we do not know), and three of them create a distinct detour to the other site of interest, we would need to add three zeros and calculating the minimum over all possible combination. Therefore, to decipher a very complex network, one needs to add extra zeros iteratively around sites with many neighbors and see if anything has changed at each point. In the example with virus data below, a loopless network emerges already after the three-way test.

The authors stress multiple times in the text that it is important to average the UEF statistic across multiple populations to reduce linkage noise. But they do not do this explicitly in their analysis of the flu data. This has to be addressed.

I now addressed this issue, as follows:

Lines 713-719:

For this aim, we compare the sequences of the first strain to the sequences of the second strain, both sampled worldwide from dozens of locations. In order to average two-site allele frequencies, f_{ij} , over virus from various local populations, we treated them as the independent multiple populations. Although they are not actually independent, but represents parts of one metapopulation, this is the best approximation one can obtain in any study working with world pandemics data

Lines 1003-1009:

It is worth noting that Influenza sequences represent a meta-population with many connected islands, not a single well-mixed population, nor completely independent populations. The approximation used in our work here is that the average over different geographic locations allows to obtain, at least, a partial average over the ensemble of independent populations. It is unclear to which extent the lack of the full ensemble average affects the estimate, but at least, we are able to compensate the residual linkage errors within that partial ensemble, something that has not been done before.

Finally, I don't quite understand how the authors estimate allele and haplotype frequencies from their data, given the fact that the sequences were sampled over multiple years. The authors appear to estimate frequencies from the entire alignment, which seems wrong because it does not represent a snapshot of a population.

We do not need a snapshot consensus,. The specific choice of the reference sequence (in our case, for time series) does not create any errors in the inferred network:

Lines 758-760:

Note that we have chosen the common consensus of the entire sequence set as the reference sequence for calculating allele frequencies. The choice of the reference sequence does not matter for the site-site correlations and hence the inferred network.

I also don't understand how the authors are confident that the mutation at site 248 that they detected actually genetically interacts with other sites, given that this mutation is present in all post-2009 N1 variants? My understanding (but I may be wrong) is that pre-2009 H1N1 lineage was outcompeted by the 2009 "swine flu" variant.

This understanding is correct, but we are not "confident", we infer. In our work, we infer how the new strain outcompetes the old strain: by developing a primary mutation with about 15 helper mutations - not passenger mutations, but 15 helper mutations.

So, couldn't it be then that the mutation at site 248 was simply present in the 2009 founder strain and is now shared by all of its descendants, without epistatically interacting with any other sites?

If that was true, then our method (which is shown analytically and by simulation to have a high fidelity) would find no significant interactions. But it does, says Fig. 2C-E.

The whole point of our paper is to infer (predict) the adaptation pathway reliably, instead of just guessing. The inferred pathway of adaptation through a fitness valley is very similar to the ones observed previously for drug-resistant or immune escape strains of HIV.

We made it more clear in Lines 776-887:

Next, we followed the procedure described above (Fig. 1C-F) to infer the intra-protein network of interactions (Fig. 2). The dependence of results on (d_v, D_w), which infers between 15 and 22 compensatory sites, originates from the unequal presentation of local subpopulations in the database. We observe that slightly different weighting gives similar results (Fig. 2C to 2E) within a plateau region in Fig. 2F. Only if one strain is strongly overrepresented, the network disappears (Figs. 2A and 2B). Below, we choose the network variant shown in Fig 2D as the "golden middle" of the set.

A primary mutation and compensatory sites. We obtain that site 248 in NA represents the primary site connected to multiple compensatory sites (Fig. 2D). Thus, our three-way method, tested in simulation and analytically, shows that the new strain that has outcompeted the old strain, only because it had a primary mutation 248 and many compensatory mutations.

What's the evidence that it is actually epistatic?

To our best knowledge, empiric evidence for or against the inferred network is not available in the literature. Thus, our method makes a new falsifiable prediction to be tested later. We made it more clear:

Lines 350-351:
We infer the primary and secondary mutations that allowed the new strain to outcompete the old strain. ....

Line 357-359:
Thus, the aim of our theoretical paper is to offer a new method of epistatic detection based on an analytic derivation, test it on simulated sequence data, apply it to a real sequence set, and make a testable prediction for a protein network. We do not aim to describe the detailed evolution of H1N1 influenza strain leaving it for the future work.

Line 1001-1007:
We did not find any empiric evidence in the literature for or against the inferred network of compensatory

mutations. Hence, we make a new testable prediction for a future experimental test. Primary site 248 can be predicted by simple alignment and is well studied experimentally. Its compensatory sites, however, are impossible to detect in vivo without our method, due to strong linkage noise. It would be very useful to compare these predictions with the results of deep mutational scanning [52, 53], which we hope will be available in the future.

Reviewer 3.

Overall:

In short, this work makes a strong contribution to our understanding of and ability to investigate the role of genetic interactions in adaptive evolution. It is certainly worthy of publication in PLoS Pathogens (I would rather see it in PLoS Biology), but I think it would benefit from the revisions I detail below.

This manuscript tackles a problem, determining the role of epistasis in adaptive evolution, of great interest to both evolutionary and molecular geneticists. Identifying interacting sites that contributed to adaptation is a challenging problem that is only now becoming accessible in the age of easy genome sequencing. The method developed and tested in this manuscript is relatively easy to understand, appears easy to implement, and offers advantages over existing methods for identifying interacting sites within adapting populations that have low to moderate genetic variation. I think the method will have applications beyond investigations of pathogen evolution, e.g. I can envision using it to identify interacting alleles in our laboratory evolution experiments (the topic of my current grant).

I thank Dr Birch for the high opinion. I am looking forward to any applications of our technique. Line numbering below is for the marked copy with traced changes.

That said, the manuscript suffers from three weaknesses that should be easy to correct in a revision. First, little to no effort is made to provide intuition for less-quantitative readers. Second, although there are mentions of having tested the limitations of the method, the results of these tests are not shown. Third, in order for this method to be used, I think the code used to conduct the analysis needs to be publicly available. I provide specific suggestions for eliminating these weaknesses below.

I did my best to address these concerns.

Specific Comments:

The intro is lacking in intuition for a non-quantitative reader. In particular, it would be good to provide more intuition for why/how the 3-way approach will eliminate stochastic noise. My guess, before I got into the Results section, was that the allele at the 3rd locus could be used to separate the data into something like the equivalent of independent experimental blocks. That guess turned out to be reasonably accurate and I think you need to provide an intuitive frame of reference, maybe like block effects, for readers in the introduction.

I added the explanation to the Introduction, Lines 263-266:

The basic idea behind this method is that demanding a majority allele at a neighbor site of a measured pair of sites, cuts the indirect detours around the pair that create false-positive interaction. In other terms, the additional condition splits the genome into independent blocks.

Line 573-579:

We observe that false-positive links are created by "detours" around pairs of sites, such as chains of indirect epistatic interactions or sited linked due to the common phylogenetic origin (red and blue curves in Fig. 1B). To break up the association created by detours, we demand that a neighbor site of the site pair of interest is 0 (better-fit, wild-type) and recalculate the correlation. If that neighbor site happens to be at the most important detour, this condition will break up or, at least, decrease the indirect correlation. Direct interactions are affected by such an additional condition to a smaller extent (if at all).

I didn't figure out until I got into the results section that the method was designed to detect pairs of sites that are (weakly) deleterious individually, but beneficial in combination. If I understood that correctly, be more explicit in the abstract and introduction about the specific kinds of interactions this method can and cannot detect.

Although we have compensatory epistasis in simulation and analysis, because this case has the strongest indirect interactions, the method applies to any type of epistasis, even negative. We made it clear in Abstract and Intro:

Lines 47-48:

These results present a simple and reliable technique to measure epistatic interactions of any sign from sequence data.

The analytic derivation in *Method* applies at any $E < 1$ and below the full compensation point, which covers all basic types of epistasis. I added an explanation:

Line 681-682:

The analytic derivation in the *Methods* applies at any $E < 1$ and below the full compensation point, which covers all basic types of epistasis.

Lines 689-683 and 1163-1167

The derivation given in the *Methods* applies at negative values of E as well, but we focus on positive E, which case is termed "diminishing returns epistasis". The reason for this choice is strong effects of epistasis and strong indirect interactions in this region. The analytic derivation applies at any $E < 1$ and below the full compensation point, which covers all basic types of epistasis.

Figure 1A also did not provide any intuition. To gain intuition for the 2-way model, I plugged in values of s1 = 0.1, s2 = 0.2, and E = 0.75 to the equations on line 162. Consider replacing Figure 1A by displaying the resulting fitness values with a landscape diagram like the one in Weinreich …

I think you are trying to use the gray box to show that an interaction with a 3rd site in the genome can affect the ability to detect the focal interaction?

If so, you can provide intuition for this issue by setting up the illustration in Fig 1A so that the allele at a third site causes a switch between those two fitness landscapes (e.g., E=0.75 or E=0). We did a similar thing in Singhal and Burch, 2020.

And, consider contrasting an example like mine with a case of E < 0.75 (maybe E=0) to give intuition for the statement on lines 257-258. I think that statement provides more intuition for the kind of epistasis this method can detect than any other statement in this manuscript.

Thank you. After so many questions, I realized that Fig. 1A is very distracting and confusing. Also, I do not use it later in the main text. Hence I eliminated it together with the entire derivation of our 2018 result in the Introduction.

Figure 1B: I didn't come away from this figure understanding your usage of indirect interactions. I did eventually puzzle it out from the text and Fig 2B. I would replace Figure 2B with statements in the text along the lines of - If locus A interacts directly with both loci B and C, there will be epistasis (i.e. E>0) between A and C, even though they do not interact directly. We call this an indirect interaction.

In the revised version, I added the proposed explanation in Lines 376-475:

We consider a simple epistatic network consisting of double arches (Fig. 1B). The network has three types of correlations: direct interactions, indirect interactions, and linkage, as shown by different colors. If the first and the second loci interact, and the second loci and the third loci interact, this leads to correlations between the allelic composition at the first and third loci, even though they do not interact, termed "indirect interaction". In addition, the first and third loci correlate due to linkage, i.e., having a common ancestor. A more complex network is discussed later on.

I prefer to keep Fig 2B (now 1B) because I need it for the explanation.

Then I would add an entirely different Fig 1B that tried to build intuition for the approach. Maybe use a cartoon phylogenetic tree (that includes lineage sorting?) to show independent evolution in isolated populations of the three loci described in Fig 1A? Or a collection of Muller diagrams (as in Lang et al)?

In the revised version, Fig. 1B is now Fig 4 and called only in the formal analytic derivation in the *Methods*. Fig. 2, which the Reviewer liked more, is now Fig. 1. I do not want to add more stuff since the paper is already sufficiently complex, and I do not want to distract the reader even more.

Lines 98-99: I don't understand the statement that allelic frequencies "are known to follow random trajectories". That makes sense to me if mutations are neutral, but the focus here is on mutations with effects on fitness.

The sentence is clarified, Lines 119-122:

Although the dependence of allelic frequencies on time is stochastic due to the combined effects of selection, random drift, and linkage, what can be said about the average allelic frequency of a given site with a given fitness effect of mutation?

Lines 144 – 148 and related methods. I find it difficult to follow the nomenclature. 1) W seems to represent individual fitness sometimes and mean fitness at other times. Use W_bar or E[W] for mean fitness, instead? 2) I think S(t) = F[W(t)] just means that S(t) is a function of W(t), but I spent time looking for F. Maybe, just write it out.

Line 148 and another earlier location: What is the distribution of W among? Individuals in the population? Genotypes in the population?

Line 155 and throughout: The use of "beneficial" and "deleterious" to distinguish between the 0 and 1 allelic states is counter-intuitive in the case of epistasis, because the benefit associated with the allele at one locus depends on the allele at the other locus. For me, the switch to consensus/majority and non-consensus/mutant gave much better intuition.

Do you mean that f_ij is proportional to the haplotype variation in the rest of the genome, **within the ij haplotype? If I have that correct, does the relationship between f_ij and # haplotypes in the rest of the genome result only from sampling effects? If so, replace "As is easy to understand" with this explanation. If not, provide an intuitive explanation.**

Lines 170-179: I don't understand the notation S(W-W_ij). What does W represent? Mean fitness? That must not be right because it doesn't explain why W-ij << W. I did not puzzle through the math to get from Eq 1 to Eq 2.

Thank you. Following so many questions and also the suggestion from Reviewer 2, I removed the entire derivation of our old results of 2018 from the Introduction. It is not used in the main text and not connected to the Methods. I did not expect it to be so distracting and confusing.

I also clarified in the *Method*s that W is always the fitness of an individual genome:

Line 1152-1153: The logarithm of the average progeny number of an individual genome, W, depends on sequence $[a_i]$ as given by…

Alleles are now introduced in Line 367-369:

We assume two possible alleles at each locus (site), 0 and 1, where 1 stands for an allele that decreases the logarithm of fitness by a fixed value $s \ll 1$ when it does not interact with any other sites.

3) Virologists often measure Shannon entropy from allele frequencies at a specific site. It is clear that is not what your entropy metric measures, but I can't puzzle out what it measures instead. The methods section really needs to explain entropy in what? What is a sequence

configuration? A haplotype? What is being clustered? Haplotypes? Individual sequences? What is a cluster configuration/shape?

Line 167: The statement starting on this line is not easy to understand. I do not understand it at all. Part of the problem is that the phrase sequence configuration is not defined. Sequence configuration = haplotype?

Now "sequence configuration" and "entropy" appear only in the *Methods*, Line 1309:

Entropy S is defined as the log number of possible sequence configurations (for example, 0111100101...)

Fig 2: Overall this is a great figure. It made it very easy for me to follow the steps in the method and to get a real sense of its power.

Thank you!

Just 2 comments that might improve it: 1) The reader encounters the acronym UFE in panel A before they encounter it in the main text.

Added to the legend:

The pairwise correlation measure UFE_{ij} is calculated from Eq. 1. The indirect links and the residual linkage are detected and filtered out by using the tri -way correlation measure, UFE_{ij0}, from Eq. 2.

2) The dashed sector doesn't add value to panel E. I would remove it.

The sector is FACS-style separation of direct and indirect. In a more complex topology, red and green dots can be closer, and you need a separating line. I now clarified its purpose in the legend to Fig. 1 (former Fig. 2):

E. Scatter plot of the three-way haplotype, $min(UFE_{ij0})$ shown against UFE_{ij} for the pairs identified in (**D**). The dashed sector corresponds to the direct interactions. The upper dashed line is the diagonal, $UFE_{ij} = UFE_{ij0}$, and the lower dashed line separates direct and indirect interactions.

Fig 3: This is a great figure, too. Is it possible to show the networks in Figs 2C, 2D, and 2F like this?

What for? The point of Fig 1 is to show the efficacy of our two-step procedure. We put a lot of work into this figure. As Dr. Birch pointed out above, it does the job.

dI'd edit the legend slightly to "… interaction for variable amino acid sites in the neuraminidase protein." And "less than dv were randomly…"

Done.

Line 298: explain why you use the minimum value of UFE_ij0. I presume it is just the most conservative choice.

Now explained, Line 584-588, Line 1236-1293:

We consider all possible connected sites, one by one, as the 0-node and calculate the minimum value of UFE_{ij0} over all possible 0-nodes, $\min(\mathrm{UFE}_{ij0})$. Finding the minimum not only detects a detour but also finds the most important detour if there is more than one. Thus, we can identify and remove false-positive links as those with a low ratio $min(UFE_{ij0})/UFE_{ij}$.

Line 314 (and somewhere earlier): I think you mean "testing" and not "training". These words are methodologically different. Be careful to use the right one.

Replaced with "testing".

Lines 335 – 341: Provide intuition for why it was necessary to compensate for unequal sampling. Why/how does unequal sampling bias the analysis?

Because it does not work otherwise. Now explained in Lines 775-787:

In order to compensate for unequal sampling from the pre-pandemic and pandemic strain, the more abundant sequences with mutation frequency per genome less than a preset value, $f < d_v$, were randomly sampled and down-weighted by a coefficient, D_w , ranging from 5% to 50%. This procedure was done to balance the number of sequences sampled between the two strains. To obtain the average pairwise haplotype frequencies, f_{ij} we repeated the resampling multiple times.

Next, we followed the procedure described above (Fig. 1C-F) to infer the intra-protein network of interactions (Fig. 2). The dependence of results on (d_v, D_w), which infers between 15 and 22 compensatory sites, originates from the unequal presentation of local subpopulations in the database. We observe that slightly different weighting gives similar results (Fig. 2C to 2E) within a plateau region in Fig. 2F. Only if one strain is strongly overrepresented, the network disappears (Figs. 2A and 2B). Below, we choose the network variant shown in Fig 2D as the "golden middle" of the set.

Results / Discussion: I wasn't satisfied by the attention paid to the sensitivity of the analysis method to various aspects of the dataset. There is some mention of such analysis, e.g. to the value of E, the amount of genetic diversity in the population, the number of isolated populations in the sample, but those analyses are not shown. Can these analyses be provided in Supplementary Material?

A possible reason for Dr Birch's dissatisfaction is misunderstanding of the genre of our work. Ours is not a data analysis project but a theoretical (modeling) work. Hence, the difference in the level of detail across different parts of the work.

The most important part, the one which was the hardest to do, and took three years to develop, is the brief analytic derivation in Methods, now with a Supplement. The second in importance is the simulation test, and the virus data are used only as a demo of what this method can do. We do not have these details on data, and I apologize for this.

In addition, I'd like to know something about the sensitivity to population demography. How much migration can there be between the ~isolated populations before the method breaks down.

Related to population demography, I would like to see a tree or trees (or Muller plots if recombination is rampant) that show flu evolution over time at the detected sites

Is 248 independently derived in many of the isolated populations, or did it move into many of the isolated populations by migration? Same question regarding the compensating mutations? Did the detected mutations all increase in frequency in more than one of the isolated populations? Did they all arise multiple times independently, or did some of them move between isolated populations by migration? In the flu data, how did the 3-way results differ from 2-way results?

These questions are very interesting, tangential to our project, and I took the liberty to add them to the *Discussion*:

Lines 1070-1076:

Regarding applications to influenza, the interesting questions to address in the future are, as follows. Is 248 independently derived in many of the isolated populations, or did it move into many of the isolated populations by migration? Same question regarding the compensating mutations? Did the detected mutations all increase in frequency in more than one of the isolated populations? Did they all arise multiple times independently, or did some of them move between isolated populations by migration? In the flu data, how did the 3-way results differ from 2-way results?

In the revised version, I also state our focus more clearly in Introduction, Lines 357-360:

Thus, the aim of our theoretical paper is to offer a new method of epistatic detection based on an analytic derivation, test it on simulated sequence data, apply it to a real sequence set, and make a testable prediction for a protein network. We do not aim to describe the evolution of H1N1 influenza strain in detail leaving it for other projects.

I also added a qualifier proof-of-principle method to the Abstract, Line 38.

Thus, the stated aim of our methodological study is **not** to understand the evolution of influenza virus before and after 2009. That will require another study, with a different focus and funding. Our goal is to propose the first, proof-of-principle method that can tease apart linkage effects from epistasis. Now that we have it, we can seriously apply it to various viruses and organisms in separate works.

Line 380: I'm back to having no intuition for what is meant by 'most disordered state'. Least LD?

Clarified in Lines 913-915:

Intuitively, the distribution of alleles between sites has a sufficient time to attain the most probable state, i.e., the state with largest number of possible sequences given fitness.

Statement on lines 404-405 re: Hamming distance should point to a supplementary figure.

The sentence is removed as redundant.

417: Define "ensemble average" and "partial ensemble average". I don't know what these terms refer to.

I clarified in Line 1008-1014:

It is worth noting that Influenza sequences represent a meta-population with many connected islands, not a single well-mixed population, nor completely independent populations. The approximation used in our work here is that the average over different geographic locations allows to obtain, at least, a partial average over the ensemble of independent populations. It is unclear to which extent the lack of the full ensemble average affects the estimate, but at least, we are able to compensate the residual linkage errors within that partial ensemble, something that has not been done before.

The Methods section needs careful copy editing, too, but I don't have the stamina to do it thoroughly. Here are some issues that need to be clarified: T_ij is not defined.

Line 1158: where $T_{ij} = 0$ or 1 is the binary matrix that shows interacting pairs.

Most of the manuscript uses the word 'interaction', but the word 'bonds' is used several times. Stick to the word 'interactions'?

"Bond" is replaced everywhere with "interaction".

The phrase 'allelic clusters' is introduced on line 507, but never defined.

Line 1297-1300

Then, we can fully characterize a genome by the numbers of interacting allelic clusters of different size, i. Each such cluster comprises i directly interacting alleles. Let k_i denote the number of clusters with i alleles and b_i interactions..

'Sequence configurations' has the same problem a few lines later

Line 1309:

Entropy S is defined as the log number of possible sequence configurations (for example, 0111100101...)

Do Ki and ki have similar meanings? If not, change ki to another letter.

Line 1154: Notation Ki replaced with ai

Where will readers be able to find the code used to conduct that analysis?

I added in Line 1525:

The core Monte-Carlo simulation code for Fig. 1 written in MATLAB is present at https://github.com/rbatorsky/hiv-recombination, which can be modified for different types of epistasis and recombination.

and in Line 1170-1174:

The core Monte-Carlo simulation code for Fig. 1 is written in MATLAB and deposited at site https://github.com/rbatorsky/hiv-recombination. It can be modified for different types of epistasis and recombination. The code for Fig 2 (data analysis) has been uploaded to https://github.com/irouzine/Pedruzzi.

The core simulation code has been used in several works and is easy to adapt to any aim. We warn Dr Birch that the second code with the final output is not user-friendly yet. Gabriele is gone. By the end of 2021, I solemnly swear to re-upload a user friendly (and better) software package, more suitable to work with the actual data. I will do it in connection with applications to influenza and SARS-CoV-2.

The manuscript would benefit from copy editing by a native English speaker. I did a lot of that below, but if PLoS will not provide copy editing, I suggest asking a colleague.

Thank you. The manuscript has been copy edited thoroughly.

Line 38: delete the last word "a"

Line 41 and throughout: Consider changing triple-way to three-way.

Line 44: change to "a large sequence database"

Line 52: change to "Interactions between genomic sites create a fitness landscape that is very…"

Line 57: consider changing to "stochastic noise, intrinsic to evolutionary processes, masking…"

Line 69: change "on" to "at"

Line 82: change from to "of"

Line 85 and elsewhere: we usually write Muller ratchet as "Muller's ratchet"

Line 132: spell out Ab as antibody

Line 134: change to "networks" plural

Line 138: add "In that work," before we simulated… (I was a little confused.)

Line 153: Change 'neglect with' to 'ignore'

Line 155: delete 'variants'

Line 189: change the second s1 to s2

Line 192: delete the comma

Line 197: change to measures of D', …

Line 212: change to 'existence of large' (delete the 'a')

Line 213: add a comma after stochastic errors

Line 226: delete second use of the word 'sequences'

Line 232: delete the word 'of'

Line 235: change to 'variant can be obtained from the old variant…'

Line 269: change to 'in the Introduction'

Line 273: change to "evolutionarily-independent"

Line 283: change fast to vast

Line 290: change 'the' to 'its'

Line 292: delete the comma

Lines 327 and 330: split aminoacid into amino acid

Line 361: change to 'after the 2009…'

Lines 372 and 384: change 'bond' to 'interaction'

Line 380: I think you mean probable instead of probably

Line 385: delete the second comma (it changes the meaning)

397: change I to It

400: change to "techniques for eliminating indirect interactions, developed…"

Line 410: change to "…at a time without the need for simultaneous optimization of the L^2/2…"

Line 413: change to "application of our method"

416: change to 'The assumption here is that average over different geographic locations…"

427: change 'less trivial' to 'more complex'

Thank you. All these corrections are introduced (and many more).
