# Supplementary material for: An evolution-based high-fidelity method of epistasis measurement: Theory and application to influenza
Source: PLoS Pathog. 2021 Jun 21;17(6):e1009669. doi: 10.1371/journal.ppat.1009669 (PMC8248644; doi:10.1371/journal.ppat.1009669)
Supplement: Attachment — Submitted filename: Rouzine 2021 Revision.pdf [file ppat.1009669.s007.pdf]

This revised manuscript is greatly improved in terms of clarity. Most of the issues I raised have been resolved and I am most appreciative of the care taken to provide greater intuition for the methods choices. I now have only one major concern, one moderate suggestion, and a few copy editing suggestions. Once the major concern has been addressed, I think the manuscript will make a strong contribution to PLoS Pathogens.

Major Concern:

In my original review, I raised questions regarding the sensitivity of the analysis method to various aspects of the dataset, in particular to population demography. It seems from the author's response that I did not explain my concern clearly. I think that the simulation data does a very nice job illustrating the power and precision of the approach for data that arise under the evolutionary and demographic regime assumed by the model (in particular, numerous isolated populations without migration between them). However, biological populations are rarely, if ever, so well behaved. Global viral pandemics certainly do not consist of numerous isolated populations with no migration between them. In that case, the simulations really should include some investigation of how well the method performs on data that are simulated using populations connected by migration.

If additional simulations are too difficult to do (e.g., because the person who conducted them moved on), some discussion about the likely effect of migration needs to be added. It seems clear that migration will make it more difficult to eliminate false positives (at the panmictic limit, the method has no value), but it will not be clear to a naïve reader how much migration can be tolerated vs. how much will render the method valueless.

Related, unless your method can answer the flu questions you added to lines 1077 – 1083, I don't see the value in adding them. I asked these questions because I thought the answers would affect the likelihood of false positives in your analysis. I think it would be better to provide readers some intuition for which of these characteristics (e.g. mutations arise many times independently vs. mutations arise once and then spread) strengthen/weaken your approach.

Moderate Suggestion:

Thank you for explaining that the data do not exist to confirm the epistatic candidates you identify in flu. In that case, I think it would strengthen your paper to add an illustration to Figure 2 or, at least, a sentence of text that describes the number and nature of the false positive interactions that were eliminated by this approach. By nature, I mean proximity to site 248 and/or whatever other metric distinguishes the false positives from the presumed true positives in your flu analysis.

Copy Editing

1. Line 255 – change cuts to reduces
2. Line 256 – splits the genome? Or, splits the sampled genomes?
3. Line 476 – change metrics to metric
4. Line 566 – change sited to sites
5. Lines 811-812 – The figure numbering changed update these pointers to Fig. 2D.

6. Figure 2. What do the different line colors mean in panels A-E? Panel F is hiding some of the site numbers on panel E.
7. Line 917 – change ‘residual effects’ to ‘false positives’?
