# Supplementary material for: An evolution-based high-fidelity method of epistasis measurement: Theory and application to influenza
Source: PLoS Pathog. 2021 Jun 21;17(6):e1009669. doi: 10.1371/journal.ppat.1009669 (PMC8248644; doi:10.1371/journal.ppat.1009669)
Supplement: Attachment — Submitted filename: Rebuttal2.pdf [file ppat.1009669.s008.pdf]

Reviewer #1:

The authors appear to have done a thorough job of responding to the reviewers' comments.

We thank the Reviewer for the favorable opinion.

Minor: The authors, in their response to my comment, state: (p. 12 - Although it seems a reasonable assumption, there should be some justification for assuming an epistatic network for NA.) "We do not assume. We infer it. We detect true interactions and measure their magnitude. I made it clear now:" Epistasis is defined as interactions among alleles that affect the phenotype. You are inferring associations among alleles, not epistasis. Also, you do not "detect true interactions" if you are inferring them.

Actually, we measure the associations and use them to predict epistatic interactions.

Clarified in Line 188:

we use our method to predict the epistatic interactions among alleles from their observed associations by isolating them from linkage effects.

It would be more convincing if you could provide independent evidence of epistasis. This is a theoretical proof-of-concept after all.

Yes, but because such an evidence does not exist at the moment, there is nothing I can do. Respectfully, this is not a data analysis paper, but a paper that develops a new inference method. The supporting arguments are analytic and simulation. The little data illustration is a prediction for the future. It is normal and useful for modeling papers to make predictions that someone can check later. Experimental tests are not our vocation.

We also added a note in Line 424:

The network has the typical appearance of a fitness valley network observed, for example, in HIV for drug-resistant mutations.

The authors state on p. 6: :Co-variation due to random linkage completely masks the epistasis signature in a population. The only way to resolve this issue is to average the haplotype frequencies over many independent populations with similar parameters under similar conditions. Without sampling multiple populations, it is not possible to infer epistasis in principle, due to the stochastic nature of phylogenetic relation of sequences. This fundamental limitation cannot be resolved by any existing or future method." Why can't this limitation be resolved by any existing or future method?

Clarified in Line 142:

As we have shown in [40], the increasingly dominant effect of linkage over epistasis in allelic associations be seen from the random divergence of independent populations in time, so that

all sequences are similar to their common ancestors, and the common ancestors move away from the origin and from each other along stochastic trajectories [40].

Also in Line 148:

The only way to resolve this issue is to average the haplotype frequencies over many independent populations with similar parameters under similar conditions. Without sampling multiple populations, it is not possible to infer epistasis in principle, due to the stochastic nature of phylogenetic relation of sequences. This fundamental limitation resulting from random phylogeny [40] cannot be resolved by any existing or future method. However, as we show below, even 200 populations may be not enough to eliminate the false-positives in a system of 40 loci.

"Why can't multiple independent populations be sampled?"

Because you need thousands, and they are never available in those numbers. Clarified in Line 153:

However, as we show below, even 200 populations may be not enough to eliminate the false-positives in a system of 40 loci.

Reviewer #2:

The authors mostly addressed my concerns related to the structure of the paper. I found the current manuscript much more readable. The additional analytical work now provided in the supplement is also a step in the right direction.

I thank the Reviewer for acknowledging our effort.

The Line numbers below are given for the marked version, in the unmarked version they are different.

However, two major concerns remain. First, the present manuscript still does not give the reader a good sense of when the method works and how well, when it is expected to fail and how. Second, the claims regarding the influenza analysis remain unsubstantiated and the analysis remains unclear. In addition, my minor concerns from the first round of review have been apparently not conveyed to the authors. I reiterate several of them below.

ROBUSTNESS OF THE METHOD. PLoS Pathogens is a journal that is read by a fairly wide audience, including practitioners who might be interested in applying the method proposed here to other viruses. So, in my opinion, a paper published in this journal needs to go a bit beyond a mere "proof of principle". Doing so is not actually too laborious. Specifically, I think that a typical PLoS Pathogens reader would want to have at least some sense of what performance to expect of the method presented here. In fact, the author's own analysis of influenza exposes the need for a sensitivity analysis. To address this concern, maybe the authors can use some quantitative metrics of performance, e.g., the rate of false positive and false negative epistatic pairs detected by their method, and report how their method

performs with respect to these metrics as evolutionary parameters vary, at least in their double arches topology shown in Figure 1B. This is fairly straightforward to do. The authors know better than me which parameters are most important to vary, but I'd certainly be interested to know at least how the strength of epistasis E and the number of independent replicates affect the performance.

To address this comment partly, I added a new paragraph in *Lines 295-300:*

"In our previous paper, we showed that averaging over dozens of population n is required to isolated epistatic links. In our example in Fig. 1, we demonstrate that, at $L = 50$ *sites and moderate population sizes, a half of false-positive links remain even after 200 populations. The present method provides 100% fidelity at* $L = 50$ *sites or less, for the number of replicate populations between 20 and 200, and epistatic strength* $E = 0.5 - 0.75$. *Performance drops sharply at* $E < 0.25$.*"*

Unfortunately, the full sensitivity analysis Reviewer wants is not straightforward at all, it is very laborious. Much more work is required to delineate the exact limits of the method. My postdoc is gone, and I cannot do it alone. Therefore, I hope the Reviewer will appreciate our paper for what it has to offer: the first method that can "see" epistasis through linkage noise. The others ignore linkage and assume the limit of strong recombination and Quasi-Linkage Equilibrium (see the review by Neher and Shraiman 2011 cited in the paper).

2. INFLUENZA CLAIMS. This part of the paper has the following two major issues. 2A. The description of the analysis of the influenza data remains unclear in several places, its organization could be streamlined, and some key pieces of information are missing. All of this makes it very difficult to understand what has been done, and makes this analysis not reproducible as described.

I did my best to address Reviewer's comments below. With respect, a detailed data analysis of the evolution of influenza is quite outside of our scope. This is only a small demonstration. Also, as I explain below in a new paragraph, our method automatically excludes the alternative scenario with hitchhiking mutations proposed by the Reviewer.

I think the following two specific points need to be addresses before I'd be comfortable recommending this paper for publication.

2A-i. Describe the analysis in more detail, provide key pieces of information in the text, make data and code available, as described in (a) through (h) below.

(a) The authors say that the sequences were split into several subsets by geographic location (LL 281-287). Into how many geographic groups have the authors actually split the data? ….. How coarse-grained are these locations? How many sequences of each type (pre-pandemic and pandemic) are in each location? As far as I can tell, this information is currently not provided anywhere. Please provide it, e.g., as a data table, and report some key summary statistics in the text.

It is a misunderstanding: we did not split the sequences by geographical location. We used the entire set to average the haplotype frequencies f_ij. Splitting them would give no advantage. I clarified the confusing sentence in Line 355, as follows:

"In order to be able to apply our method, we assumed that various local populations are nearly independently evolving, and migration between them is very slow".

(b) Was the frequency cutoff of 5% applied to each location separately or is it 5% of the total number of sequences, i.e., 8440 * 0.05 = 422? I suspect it is the latter. In this case, please state this explicitly.

To the total, as it is clear now.

(c) Please provide the estimated consensus sequence as well as the binarized individual sequences as a supplementary data files.
(e) Please show the distribution of mutant allele frequencies as a figure and please provide an estimate of the fraction of sequences around each peak.
(f) I also would very much like to see a phylogeny showing how the pre-pandemic and pandemic clades are related to each other.

I do not have these data, sorry, and we did not need them for the application of our method.

Or at the very least provide the code and the raw sequence data file that you used that would generate all these intermediate data files.

The code and raw data have been deposited, see Lines 611-612, although it is not required for reading the revised text.

Specifically, I would like to see how many mutations separate the pandemic clade from the rest of the pre-pandemic variants.

The segregating sites are more numerous than about 15 inferred epistatic mutations. Clarified in Line 377:

The old and the new strain differed in NA protein in about 100 sites, which is 20% of its length.

This information is critical for understanding how the authors arrive at their epistasis network for site 248 later on.

Not really (see below).

(g) Rather than saying "multiple times", please specify how many times exactly has the resampling been performed (L 314).

Added in Line 413: *"we repeated resampling 200 times.*

(h) Please provide the estimated values of the UFE and conditional UFE statistics for each location for each pair of sites in a supplementary data file.

As we made it now clear in the figure caption, for the NA protein, the strength of all links shown is *UFE > 0.5*. We did not keep the exact values. For the simulation in Fig 1, the exact numbers are shown at the links.

2A-ii. Streamline this part of the paper.

LL 281-287. I don't understand what the purpose of this paragraph is. Is it a description of what is being done or an introduction into what will be described next? The next paragraph describes the processing of the data, so I infer that the current paragraph is introductory. Then it is unnecessary and confusing. The previous paragraph gives enough background information, and diving right into a brief description of what is being done would be preferable. The current paragraph also mentions how sequences were divided among geographic locations, which is not explained in more details anywhere else, as far as I could tell.

The purpose of the paragraph is to describe what we are about to do and to state the chief assumptions. Since one sentence confused the Reviewer into thinking we split populations, I clarified it now in Line 355:

"In order to apply our method, we assumed that various local populations are nearly independently evolving, and migration between them is very slow".

LL 288-300. IMO, this paragraph largely belongs to the Methods section and also requires more details, as discussed above.

We swapped it with the paragraph from *Methods* which fits results better. Now it reads:

Line 360: We have used about 8000 sequences found in the cited database for the period 2000-2010 from various geographic locations. Our goal was to understand the difference between the strains before and after pandemic of 2009, which have 80% of mutual homology. We wished to infer only the epistatic sub-network related to that difference. For this end, we compared worldwide samples of sequences from the two strains. We randomly sampled similar amounts of sequences from the first and second strains, and re-sampled them several hundred times. We also checked the robustness of the results to the exact sampling size (Fig. 1F).

2B. There is currently not enough support for the main result that the mutation at site 248 genetically interacts with other sites. I once again ask the authors to explain how they reject the following hypothesis. Suppose that all pandemic N1 variants carry 15 to 22 mutations (including a mutation at site 248) that differentiate all these variants from all pre-pandemic variants. In this case, there is simply no information about the epistatic interactions between these 15 to 22 sites. All these mutations could be hitchhikers. We only know that they form a single haplotype which is sweeping to fixation. The argument that "our three-way method, tested in simulation and analytically, shows…" is not sufficient because neither the simulations nor the analytical calculations have explored a scenario where a new strain with dozens of new mutations invades a population. This is not a quasi-

equilibrium travelling wave scenario that was at least to some extent explored in the first part of the paper. So, the fact that the conditional UFE method reveals some significant pairs in this analysis is not sufficient to convince me that site 248 is actually involved in any epistatic interactions, let alone the claim that "the new strain that has outcompeted the old strain, only because it had a primary mutation 248 and many compensatory mutations."

To address this important concern, I gave a simple explanation, which does not appeal to traveling wave and shows that our method does not detect hitchiking mutations, Line 432-467:

One might ask whether these inferred mutations in Fig. 2 are simply a driving mutation with hitchhiker mutations present in the invading strain. As easy to understand, in this case, our method would give zero signal instead of the network inferred (Fig. 2). To make this fact obvious, we consider an extreme example, where the population represents a mix of the uniform old and uniform new strain. We focus on the part of the genome where the two strain differ, for example, 101100… and 010011 …, respectively. These differences can be either due to driving or hitchhiking mutations. Note that, for any pair of these sites, only two haplotypes f_{ij} *out of the four are present. For example, for the first and second sites, we have haplotypes 10 and 01. Hence, when calculating correlation* UFE_{ij} *and* UFE_{ij0} *in Eqs. 1 and 2, these pairs are excluded from analysis due to the cutoff condition,* $f_{ij} > 0.05$. *Thus, by design, our method does not measure the difference between strains, but only a specific type of association between the fluctuations of alleles that is caused by epistasis.*

A smaller but still important issue is that the phase diagram in Figure 4F shows that the number of detected interacting site pairs is quite sensitive to the choice of the resampling parameters. But the authors do not specify how these parameters should be chosen. They simply declare by fiat that choices A and B are incorrect whereas choices C, D, E are correct. I don't understand what justifies this choice.

Robustness. I explained our choice better in Line 420:

We choose cases C, D, E based on their robustness with respect to the two sampling parameters seen as the broad plateau in the 2D diagram, see panels C, D, E. These cases correspond to a roughly equal amount of each strain. If one strain is strongly overrepresented, the network disappears (Figs. 2A and 2B). Below, we choose the network variant shown in Fig 2D as the "golden middle" of the set.

Minor. Throughout the text. It is stylistically preferable to avoid loose jargon such as "detour", "kills indirect interactions" and others. "Detour" is used particularly often. I suggest that the authors either define what they mean by "detour" more precisely or simply replace it with something more precise like "indirect interaction" or "spurious interaction".

Now defined in Lines 161-162:

The basic idea behind this method is that demanding a majority allele at a neighbor site of a measured pair of sites interrupts a "detour", i.e., a path along interacting sites that creates a false-positive interaction for the pair of interest.

LL 190. How is the UFE_{ij} statistic (or the UFE_{ij0}) evaluated if either f_{01} or f_{10} are zero?

Thank you, this is an important point, which also addresses a Reviewer's concern above. We exclude such pairs and keep only the sites where all four haplotypes are present. Added in Line 249:

Because the logarithms in Eq. 1 diverge when one of the four haplotype frequencies is zero, we consider only site pairs such that all four f_{ij} *are larger than* f_{cut}*, where* $f_{cut} \ll 1$ *is a low cutoff, which is set below at* $f_{cut} = 0.05$*.*

LL 237-248. Please provide in the Results section the expressions for the UFE_{ij} and UFE_{ij0} statistics for epistatic and non-epistatic pairs obtained in the analytical calculations. I think it's fine to relegate the calculations themselves to the Methods section. But the reader needs to see some substantiation for the statements made in the paragraph.

I copied the equations to Results, Lines 304 and 305.

$$\mathrm{UFE}_{ij} \approx \frac{1}{4(1-E)}$$

$$\mathrm{UFE}_{ij0} \approx 0$$

LL 245. It seems that calling this type of epistasis "diminishing returns" is not quite accurate ("diminishing returns epistasis" usually refers to beneficial mutations), and adding this label doesn't add any value to this discussion. I suggest removing it.

It depends on the reference frame. We study the accumulation of beneficial alleles (or reversion of deleterious alleles, which is the same), and two mutations together give less fitness than two separately, so the term is justified from that point of view. Even though the expression for *W* is written formally in terms of deleterious alleles against the best fit background.

LL 357-359. This statement "We note that Influenza virus has been shown to map to the traveling wave theory …" is somewhat misleading. Yes, antigenic drift in influenza is reasonably well described by the travelling wave theory. However, this is not the scenario that the authors tackle here. The pandemic 2009 flu is a relatively distant strain of H1N1 that invaded the resident H1N1 strain. So, it's not just a single travelling wave situation, as mentioned above. Please rephrase.

We observe not a uniform invading strain, which would predict zero epistasis in our method, see above, but a two-component traveling wave. The genetic distance between them is not relevant, relevant are the slow dynamics.

I clarified this point in Line 370:

In terms of travelling wave, that implies that we have two traveling waves with overlapping fitness distribution, one is gradually waning over several years and replacing the other.

L 427. Should there be a + sign in front of the second sum? Otherwise, I do not see how E = 1 represents compensation.

Thank you. The typo in Eq. 3 is fixed.

L 441. What exactly is f0?

Explained in Line 599: *"with the initial frequency of deleterious alleles,* $f_0 = 0.45$*"*

L 441. Do U and μL represent the same quantity?

μL is now U.

L 446. What does "epistatically isolated" mean?

Changed in Line 632: *"they do not interact with other sites".*

LL 454-457. I think this statement requires some qualifications. I think the authors mean that the evolving population reaches the maximum-entropy state at the given fitness level with respect to the polymorphic sites. There could be other genotypes that have the same level of fitness but would requires flipping some sites that are fixed in the population.

Correct. Added in Line 664: *The evolving population reaches the maximum-entropy state at the given fitness level with respect to the polymorphic sites.*

Section "Analytic test of the method" (LL 479-523). I am confused. Are these calculations for the "double arches" topology shown in Figure 1 or for the "triple arches" topology shown in Figure 4. These paragraphs refer to both figures. See my related comment to Figure 4 below. Please resolve this confusion.

Double arches. The extra arch is removed, thank you.

L 489. "interaction s" remove the space.

Fixed.

LL 510-511. I still don't follow how the expression for UFEij is derived from Eq (9). It seems some approximations are made. Please state them explicitly.

Clarified in Line 679 before Eq. 9:

When epistatic interaction is sufficiently strong, as given by the condition E > 1/2, triplets dominate numerically over single alleles and doubles, as given by $f_1 \ll f_2 \ll f_3$ *(Eq. 7). From Eqs. 7 and 8, using these strong inequalities, we can approximate the haplotype frequencies as*

LL 513-514. Please provide an expression for the UFE for a truly epistatic pair so that the expressions for UFE for epistatic and non-epistatic pairs can be directly compared.

Added to Line 625:

$$UFE_{ij} \approx \frac{1}{4(1-\ E)} \qquad (10)$$

For directly interacting sites 1 and 2 (Fig. 1), we previously obtained $f_{11}^{dir} \approx f_1^{3-4E}, f_{10}^{dir} \approx \frac{1}{3} f_1^{2-2E}$ *[see [44], Supplement, Eqs. (3.29) and (3.30)]. This gives the same result, Eq. 10. We observe that the indirect covariance between sites 1 and 3 is as strong as for directly interacting sites.*

LL 517-518. And the same for the UFE_{ij0}. Direct comparison of conditional UFE for both types of pairs would also be helpful.

Added to Line 633:

$$f_{101} \approx f_1^2,\ f_{100} \approx f_1 \qquad (11\ )$$
$$UFE_{ij0} \approx 0.$$

L 541-544: What is a guide tree and how is it used? It seems that it is used to generate the consensus sequence, which is then used to binarize each amino acid residue in each sequence. But how the consensus sequence is obtained is not clear from this description.

I removed the mysterious sentence in Line 753 and explained: *"The obtained consensus, defined as the most frequent variant"*

Figure 4.
(1) This looks like "triple arches" to me, not "double arches", so it is a different topology than the one that the authors explore numerically in Figure 1. I pointed this out in my first review, but this has not been addressed. Please resolve this.

The lower arch is removed, thank you for noticing.

(2) What does "active interaction" mean?

Clarified: *Interactions between the existing deleterious alelles are shown in red.*

Reviewer #3:

This revised manuscript is greatly improved in terms of clarity. Most of the issues I raised have been resolved and I am most appreciative of the care taken to provide greater intuition for the methods choices. I now have only one major concern, one moderate suggestion, and a few copy editing suggestions. Once the major concern has been addressed, I think the manuscript will make a strong contribution to PLoS Pathogens.

Thank you for the very favorable opinion.

Major Concern:

In my original review, I raised questions regarding the sensitivity of the analysis method to various aspects of the dataset, in particular to population demography. It seems from the author's response that I did not explain my concern clearly. I think that the simulation data does a very nice job illustrating the power and precision of the approach for data that arise under the evolutionary and demographic regime assumed by the model (in particular, numerous isolated populations without migration between them). However, biological populations are rarely, if ever, so well behaved. Global viral pandemics certainly do not consist of numerous isolated populations with no migration between them. In that case, the simulations really should include some investigation of how well the method performs on data that are simulated using populations connected by migration.

If additional simulations are too difficult to do (e.g., because the person who conducted them moved on),

This is exactly the case, thank you for understanding. Also, modeling metapopulation evolution requires a completely different approach and software.

some discussion about the likely effect of migration needs to be added. It seems clear that migration will make it more difficult to eliminate false positives (at the panmictic limit, the method has no value), but it will not be clear to a naïve reader how much migration can be tolerated vs. how much will render the method valueless.

This is a super-hard problem, especially in the presence of linkage and selection! For now, I added a paragraph in Lines 543-555:

The application of our technique to the metapopulation of influenza is based on the assumption that migration between local populations ("demes") is sufficiently slow, so that their evolution along stochastic trajectories remains mostly independent. The standard criterion of independence is that, for the genomic region of interest, the migration rate from directly connected demes is much smaller than the mutation rate per region. In the opposite limit when migration is very fast, the entire metapopulation is well-mixed, and the method becomes useless. There exists a large intermediate interval of migration rates when the neighboring connected demes form well-mixed clusters, and the population represents a large number of such independent clusters, so the method still applies, except the linkage noise is increasing as their number is decreasing. When migration is so fast that a new best-fit genome arising in the metapopulation spreads faster to any deme than the local production of best-fit genomes by mutation, we have the panmixia case and the method ceases to apply.

Related, unless your method can answer the flu questions you added to lines 1077 – 1083, I don't see the value in adding them. I asked these questions because I thought the answers would affect the likelihood of false positives in your analysis. I think it would be better to provide readers some intuition for which of these characteristics (e.g. mutations arise many

times independently vs. mutations arise once and then spread) strengthen/weaken your approach.

The results for fitness landscape are independent on the details of the evolutionary trajectory, except that we need the quasi-independence of local populations, see above. Hence, following the suggestion, I removed these sentences.

Moderate Suggestion:

Thank you for explaining that the data do not exist to confirm the epistatic candidates you identify in flu. In that case, I think it would strengthen your paper to add an illustration to Figure 2 or, at least, a sentence of text that describes the number and nature of the false positive interactions that were eliminated by this approach. By nature, I mean proximity to site 248 and/or whatever other metric distinguishes the false positives from the presumed true positives in your flu analysis.

I added this statement to Line 470:

"Unlike the epistatic links in Fig. 2 and 3, false positives are not linked to any specific biology or proximity to site 248. Linkage is indiscriminatory in this sense, because it is a trivial consequence of the stochastic phylogeny. Almost every pair of diverse sites in the database is a potential false positive interaction, see Fig 1C as an illustration."

Copy Editing

1. **Line 255 – change cuts to reduces.** Now "*interrupts*".
2. **Line 256 – splits the genome? Or, splits the sampled genomes?** Splits the genome (in the general sense)
3. **Line 476 – change metrics to metric.** Done.
4. **Line 566 – change sited to sites.** Done.

6. **Lines 811-812 – The figure numbering changed update these pointers to Fig. 2D.** Done.
7. **Figure 2. What do the different line colors mean in panels A-E?** Added: *(A-E) Color codes for location in the protein*
8. **Panel F is hiding some of the site numbers on panel E**. The hidden numbers are the same as in the panel D, so I did not repeat them.
9. **Line 917 – change 'residual effects' to 'false positives'?** Changed to *"residual false positives".*
