# Supplementary material for: An evolution-based high-fidelity method of epistasis measurement: Theory and application to influenza
Source: PLoS Pathog. 2021 Jun 21;17(6):e1009669. doi: 10.1371/journal.ppat.1009669 (PMC8248644; doi:10.1371/journal.ppat.1009669)
Supplement: Attachment — Submitted filename: Rouzine 2021.docx [file ppat.1009669.s005.docx]

**Overall:**

In short, this work makes a strong contribution to our understanding of and ability to investigate the role of genetic interactions in adaptive evolution. It is certainly worthy of publication in PLoS Pathogens (I would rather see it in PLoS Biology), but I think it would benefit from the revisions I detail below.

This manuscript tackles a problem, determining the role of epistasis in adaptive evolution, of great interest to both evolutionary and molecular geneticists. Identifying interacting sites that contributed to adaptation is a challenging problem that is only now becoming accessible in the age of easy genome sequencing. The method developed and tested in this manuscript is relatively easy to understand, appears easy to implement, and offers advantages over existing methods for identifying interacting sites within adapting populations that have low to moderate genetic variation. I think the method will have applications beyond investigations of pathogen evolution, e.g. I can envision using it to identify interacting alleles in our laboratory evolution experiments (the topic of my current grant).

That said, the manuscript suffers from three weaknesses that should be easy to correct in a revision. First, little to no effort is made to provide intuition for less-quantitative readers. Second, although there are mentions of having tested the limitations of the method, the results of these tests are not shown. Third, in order for this method to be used, I think the code used to conduct the analysis needs to be publicly available. I provide specific suggestions for eliminating these weaknesses below.

**Specific Comments:**

1. The intro is lacking in intuition for a non-quantitative reader. In particular, it would be good to provide more intuition for why/how the 3-way approach will eliminate stochastic noise. My guess, before I got into the Results section, was that the allele at the 3^rd^ locus could be used to separate the data into something like the equivalent of independent experimental blocks. That guess turned out to be reasonably accurate and I think you need to provide an intuitive frame of reference, maybe like block effects, for readers in the introduction.
2. I didn’t figure out until I got into the results section that the method was designed to detect pairs of sites that are (weakly) deleterious individually, but beneficial in combination. If I understood that correctly, be more explicit in the abstract and introduction about the specific kinds of interactions this method can and cannot detect.
3. Figure 1A also did not provide any intuition. To gain intuition for the 2-way model, I plugged in values of s1 = 0.1, s2 = 0.2, and E = 0.75 to the equations on line 162. Consider replacing Figure 1A by displaying the resulting fitness values with a landscape diagram like the one in Weinreich … And, consider contrasting an example like mine with a case of E < 0.75 (maybe E=0) to give intuition for the statement on lines 257-258. I think that statement provides more intuition for the kind of epistasis this method can detect than any other statement in this manuscript. I think you are trying to use the gray box to show that an interaction with a 3^rd^ site in the genome can affect the ability to detect the focal interaction? If so, you can provide intuition for this issue by setting up the illustration in Fig 1A so that the allele at a third site causes a switch between those two fitness landscapes (e.g., E=0.75 or E=0). We did a similar thing in Singhal and Burch, 2020.
4. Figure 1B: I didn’t come away from this figure understanding your usage of indirect interactions. I did eventually puzzle it out from the text and Fig 2B. I would replace Figure 2B with statements in the text along the lines of - If locus A interacts directly with both loci B and C, there will be epistasis (i.e. E>0) between A and C, even though they do not interact directly. We call this an indirect interaction. Then I would add an entirely different Fig 1B that tried to build intuition for the approach. Maybe use a cartoon phylogenetic tree (that includes lineage sorting?) to show independent evolution in isolated populations of the three loci described in Fig 1A? Or a collection of Muller diagrams (as in Lang et al)?
5. Lines 98-99: I don’t understand the statement that allelic frequencies “are known to follow random trajectories”. That makes sense to me if mutations are neutral, but the focus here is on mutations with effects on fitness.
6. Lines 144 – 148 and related methods. I find it difficult to follow the nomenclature. 1) W seems to represent individual fitness sometimes and mean fitness at other times. Use W_bar or E[W] for mean fitness, instead? 2) I think S(t) = F[W(t)] just means that S(t) is a function of W(t), but I spent time looking for F. Maybe, just write it out. 3) Virologists often measure Shannon entropy from allele frequencies at a specific site. It is clear that is not what your entropy metric measures, but I can’t puzzle out what it measures instead. The methods section really needs to explain entropy in what? What is a sequence configuration? A haplotype? What is being clustered? Haplotypes? Individual sequences? What is a cluster configuration/shape?
7. Line 148 and another earlier location: What is the distribution of W among? Individuals in the population? Genotypes in the population?
8. Line 155 and throughout: The use of “beneficial” and “deleterious” to distinguish between the 0 and 1 allelic states is counter-intuitive in the case of epistasis, because the benefit associated with the allele at one locus depends on the allele at the other locus. For me, the switch to consensus/majority and non-consensus/mutant gave much better intuition.
9. Line 167: The statement starting on this line is not easy to understand. I do not understand it at all. Part of the problem is that the phrase sequence configuration is not defined. Sequence configuration = haplotype? Do you mean that f_ij is proportional to the haplotype variation in the rest of the genome, **within the ij haplotype**? If I have that correct, does the relationship between f_ij and # haplotypes in the rest of the genome result only from sampling effects? If so, replace “As is easy to understand” with this explanation. If not, provide an intuitive explanation.
10. Lines 170-179: I don’t understand the notation S(W-W_ij). What does W represent? Mean fitness? That must not be right because it doesn’t explain why W-ij << W. I did not puzzle through the math to get from Eq 1 to Eq 2.
11. Fig 2: Overall this is a great figure. It made it very easy for me to follow the steps in the method and to get a real sense of its power. Just 2 comments that might improve it: 1) The reader encounters the acronym UFE in panel A before they encounter it in the main text. 2) The dashed sector doesn’t add value to panel E. I would remove it.
12. Fig 3: This is a great figure, too. Is it possible to show the networks in Figs 2C, 2D, and 2F like this? I’d edit the legend slightly to “… interaction for variable amino acid sites in the neuraminidase protein.” And “less than dv were randomly…”
13. Line 298: explain why you use the minimum value of UFE_ij0. I presume it is just the most conservative choice.
14. Line 314 (and somewhere earlier): I think you mean “testing” and not “training”. These words are methodologically different. Be careful to use the right one.
15. Lines 335 – 341: Provide intuition for why it was necessary to compensate for unequal sampling. Why/how does unequal sampling bias the analysis?
16. Results / Discussion: I wasn’t satisfied by the attention paid to the sensitivity of the analysis method to various aspects of the dataset. There is some mention of such analysis, e.g. to the value of E, the amount of genetic diversity in the population, the number of isolated populations in the sample, but those analyses are not shown. Can these analyses be provided in Supplementary Material?
17. In addition, I’d like to know something about the sensitivity to population demography. How much migration can there be between the ~isolated populations before the method breaks down.
18. Related to population demography, I would like to see a tree or trees (or Muller plots if recombination is rampant) that show flu evolution over time at the detected sites. Is 248 independently derived in many of the isolated populations, or did it move into many of the isolated populations by migration? Same question regarding the compensating mutations? Did the detected mutations all increase in frequency in more than one of the isolated populations? Did they all arise multiple times independently, or did some of them move between isolated populations by migration?
19. In the flu data, how did the 3-way results differ from 2-way results?
20. Line 380: I’m back to having no intuition for what is meant by ‘most disordered state’. Least LD?
21. Statement on lines 404-405 re: Hamming distance should point to a supplementary figure.
22. 417: Define “ensemble average” and “partial ensemble average”. I don’t know what these terms refer to.
23. The Methods section needs careful copy editing, too, but I don’t have the stamina to do it thoroughly. Here are some issues that need to be clarified: T_ij is not defined. Most of the manuscript uses the word ‘interaction’, but the word ‘bonds’ is used several times. Stick to the word ‘interactions’? The phrase ‘allelic clusters’ is introduced on line 507, but never defined. ‘Sequence configurations’ has the same problem a few lines later. Do Ki and ki have similar meanings? If not, change ki to another letter.
24. Where will readers be able to find the code used to conduct that analysis?

**The manuscript would benefit from copy editing by a native English speaker. I did a lot of that below, but if PLoS will not provide copy editing, I suggest asking a colleague.**

25. Line 38: delete the last word “a”
26. Line 41 and throughout: Consider changing triple-way to three-way.
27. Line 44: change to “a large sequence database”
28. Line 52: change to “Interactions between genomic sites create a fitness landscape that is very…”
29. Line 57: consider changing to “stochastic noise, intrinsic to evolutionary processes, masking…”
30. Line 69: change “on” to “at”
31. Line 82: change from to “of”
32. Line 85 and elsewhere: we usually write Muller ratchet as “Muller’s ratchet”
33. Line 132: spell out Ab as antibody
34. Line 134: change to “networks” plural
35. Line 138: add “In that work,” before we simulated… (I was a little confused.)
36. Line 153: Change ‘neglect with’ to ‘ignore’
37. Line 155: delete ‘variants’
38. Line 189: change the second s1 to s2
39. Line 192: delete the comma
40. Line 197: change to measures of D’, …
41. Line 212: change to ‘existence of large’ (delete the ‘a’)
42. Line 213: add a comma after stochastic errors
43. Line 226: delete second use of the word ‘sequences’
44. Line 232: delete the word ‘of’
45. Line 235: change to ‘variant can be obtained from the old variant…’
46. Line 269: change to ‘in the Introduction’
47. Line 273: change to “evolutionarily-independent”
48. Line 283: change fast to vast
49. Line 290: change ‘the’ to ‘its’
50. Line 292: delete the comma
51. Lines 327 and 330: split aminoacid into amino acid
52. Line 361: change to ‘after the 2009…’
53. Lines 372 and 384: change ‘bond’ to ‘interaction’
54. Line 380: I think you mean probable instead of probably
55. Line 385: delete the second comma (it changes the meaning)
56. 397: change I to It
57. 400: change to “techniques for eliminating indirect interactions, developed…”
58. Line 410: change to “…at a time without the need for simultaneous optimization of the L^2/2…”
59. Line 413: change to “application of our method”
60. 416: change to ‘The assumption here is that average over different geographic locations…”
61. 427: change ‘less trivial’ to ‘more complex’?
